# A Guyon's canal ganglion presenting as occupational overuse syndrome: A case report

**DOI:** 10.1186/1749-7221-3-4

**Published:** 2008-02-12

**Authors:** Jeffrey CY Chan, William H Tiong, Michael J Hennessy, John L Kelly

**Affiliations:** 1 Department of Plastic, Reconstructive and Hand Surgery, University Hospital Galway, Galway, Ireland; 2 Department of Neurology, University Hospital Galway, Galway, Ireland

## Abstract

**Background:**

Occupational overuse syndrome (OOS) can present as Guyon's canal syndrome in computer keyboard users. We report a case of Guyon's canal syndrome caused by a ganglion in a computer user that was misdiagnosed as OOS.

**Case presentation:**

A 54-year-old female secretary was referred with a six-month history of right little finger weakness and difficulty with adduction. Prior to her referral, she was diagnosed by her general practitioner and physiotherapist with a right ulnar nerve neuropraxia at the level of the Guyon's canal. This was thought to be secondary to computer keyboard use and direct pressure exerted on a wrist support. There was obvious atrophy of the hypothenar eminence and the first dorsal interosseous muscle. Both Froment's and Wartenberg's signs were positive. A nerve conduction study revealed that both the abductor digiti minimi and the first dorsal interosseus muscles showed prolonged motor latency. Ulnar conduction across the right elbow was normal. Ulnar sensory amplitude across the right wrist to the fifth digit was reduced while the dorsal cutaneous nerve response was normal. Magnetic resonance imaging of the right wrist showed a ganglion in Guyon's canal. Decompression of the Guyon's canal was performed and histological examination confirmed a ganglion. The patient's symptoms and signs resolved completely at four-month follow-up.

**Conclusion:**

Clinical history, occupational history and examination alone could potentially lead to misdiagnosis of OOS when a computer user presents with these symptoms and we recommend that nerve conduction or imaging studies be performed.

## Introduction

Occupational overuse syndrome (OOS) describes a range of ergonomic injuries that result from repetitive demand over time and may be induced by occupation, recreational or leisure activity [1,2]. Guyon's canal syndrome is a well described ulnar nerve entrapment syndrome at the wrist level, and OOS can present as Guyon's canal syndrome in computer keyboard users. Various aetiologies such as trauma, ganglia, ulnar artery aneurysm, anomalous muscle, lipoma, rheumatoid arthritis and fracture of carpal bones have been reported [3]. We report a case of Guyon's canal syndrome caused by a ganglion in a computer user that was misdiagnosed as OOS.

## Case history

A 54-year-old female secretary was referred with a six-month history of right little finger weakness and difficulty with adduction. She also complained of difficulty with pronation especially when turning a key and found that her right wrist felt subjectively weak.

Four weeks prior to her referral, she was diagnosed by her general practitioner and physiotherapist with a right ulnar nerve neuropraxia at the level of the Guyon's canal. This was thought to be secondary to using a computer keyboard and direct pressure exerted on a wrist support. A provisional diagnosis of occupational overuse syndrome was made. The patient was advised to avoid prolonged wrist extension while typing and to avoid the use of a wrist support. Four weeks later, she consulted a hand surgeon about her problem.

**Figure 1:**
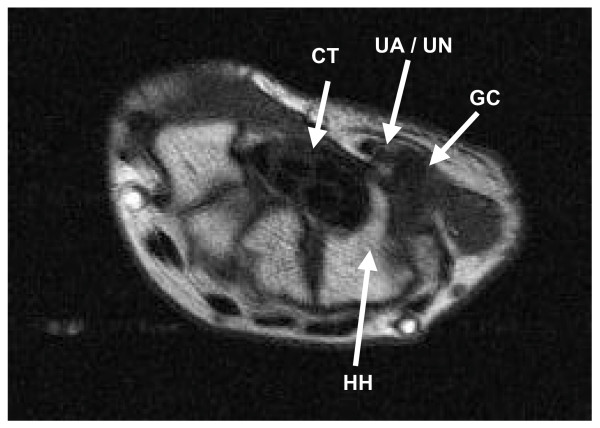
MRI scan of the right wrist showing a ganglion cyst (GC) in the region of the ulnar artery and nerve (UA/UN) medial to the right carpal tunnel (CT) and hook of hamate (HH).

On examination, there was obvious atrophy of the hypothenar eminence and the first dorsal interosseous muscle. Both Froment's and Wartenberg's signs were positive. Tinel's sign was absent and no mass was palpable in the wrist, forearm or elbow. There was no sensory deficit. A nerve conduction study revealed that both the abductor digiti minimi and the first dorsal interosseus muscles showed prolonged motor latency (Table 1). Only rare fibrillations were detected on the electromyogram of the first dorsal interosseous muscle. Ulnar conduction across the right elbow was normal. Ulnar sensory amplitude across the right wrist to the fifth digit was reduced while the ulnar dorsal cutaneous nerve response was normal (Table 2). There were no symptoms suggestive of cervical radiculopathy or brachial plexopathy. Radiographs of the cervical spine and the right hand were normal. Magnetic resonance imaging of the right wrist showed a ganglion cyst arising from the wrist and penetrating the ulnar collateral ligament, medial to the carpal tunnel and the hook of hamate (Figure 1).

**Table 1:** Nerve conduction parameters (motor components) showing prolonged motor latency of both the abductor digiti minimi and the first dorsal interosseus muscles.

MOTOR NERVES	Latency (ms)	Amplitude (mV)	Conduction Velocity (m/s)	Amplitude% (%)
Right Median				
Wrist-APB	2.6	7.1		

Right Ulnar				
Wrist-ADM	4.0	2.7		
Below Elbow-Wrist	8.2	2.4	50.0	-11
Above Elbow-Wrist	10.0	2.8	69.4	15
Wrist-FDI	6.0	0.8		-73

**Table 2:** Never conduction parameters (sensory components) showing normal ulnar dorsal cutaneous nerve response while the ulnar sensory amplitude across the right wrist to the fifth digit was reduced.

SENSORY NERVES	Latency (ms)	Amplitude (μV)
Right C.T.S.		
F2-Wrist	3.0	26
F5-Wrist	2.3	3.1

Right UDCN	1.85	16

Decompression of the Guyon's canal was performed under general anaesthesia. A ganglion measuring 1.1 × 0.4 × 0.3 cm was identified and excised. Histological examination showed a multi-cystic lesion that was composed of a dense collagenous wall lined in part by flattened synovial cells, confirming a ganglion cyst. The patient's symptoms and signs completely resolved at four month follow-up.

## Discussion

In the absence of typical symptoms, vague hand symptoms are often referred to physiotherapists for a period of conservative non-surgical management. A trial of muscle strengthening exercise, splintage or activity avoidance is often suggested to relieve these symptoms.

OOS is defined physiologically as repetitive microtrauma that is sufficient to overwhelm the tissues' ability to adapt [4]. OOS is an umbrella term for work-related disorders that develop as a result of repetitive movements, awkward postures or abnormal force due to ergonomic hazards. Diagnosis is obtained through careful medical and occupational history, clinical examination and exclusion of non-occupational diseases [5].

Non-occupational disorders are differential diagnoses when OOS is suspected, but in this case, the suggestive occupational history had misguided the judgements of both the general practitioner and the physiotherapist. In this case, even though distal ulnar neuropathy was correctly identified, the cause of the problem was attributed to OOS because of the history of frequent and repetitive computer keyboard use and the use of a wrist support. Non-specific symptoms that would support OOS such as difficulty in forearm pronation and wrist motion in this patient may also influence the misdiagnosis. Hence, a period of physiotherapy with activity avoidance was suggested based on the initial clinical impression. In fact, frequent and regular pressure of the ganglion against the ulnar nerve during keyboard use may have resulted in symptoms that would not have otherwise manifested until later. In hindsight, this was supported by the finding that the ganglion was rather small when compared with those documented in the literature [6-8].

Guyon's canal syndrome due to occupational overuse has been attributed to prolonged flexion or extension of the wrist and repeated pressure on the hypothenar eminence [5]. Guyon's canal syndrome due to occupational trauma can be improved by behavioural modification [9]. Identification of a treatable cause and early intervention can lead to resolution of symptoms and help to preserve function [4]. It has been reported that approximately 10% of computer users who have work-related symptoms were found to have positive Tinel's sign over the Guyon's canal [10]. The occupational history and lack of specific criteria for diagnosis of OOS makes it difficult to exclude a treatable lesion without the aid of further investigations.

Clinical history, occupational history and examination alone could potentially lead to misdiagnosis of OOS when a computer user presents with Guyon's canal syndrome, as we have illustrated here. Therefore, we recommend that nerve conduction or imaging studies be performed in patients presenting with similar complaints.

## Abbreviations

ADM – abductor digiti minimi

APB – abductor pollicis brevis

FDI – First dorsal interosseous

F2 – Second finger

F5 – Fifth finger

UDCN – Ulnar dorsal cutaneous nerve

## Competing interests

The author(s) declare that they have no competing interests.

## Authors' contributions

JC and JK conceived the case report and interpreted the data. JC performed all pertinent literature review on the subject and drafted the manuscript. JK performed the patient's surgery. WT helped to conceive the case report and participated in data analysis. MH performed and interpreted the patient's nerve conduction tests in the neurology service. All authors approved the final manuscript.
